# Deciphering the Intercellular Communication Between Immune Cells and Altered Vascular Smooth Muscle Cell Phenotypes in Aortic Aneurysm From Single-Cell Transcriptome Data

**DOI:** 10.3389/fcvm.2022.936287

**Published:** 2022-06-28

**Authors:** Genmao Cao, Zhengchao Lu, Ruiyuan Gu, Xuezhen Xuan, Ruijing Zhang, Jie Hu, Honglin Dong

**Affiliations:** ^1^Department of Vascular Surgery, The Second Hospital of Shanxi Medical University, Taiyuan, China; ^2^Department of Nephrology, The Second Hospital of Shanxi Medical University, Taiyuan, China

**Keywords:** aortic aneurysm, immune cells, VSMC phenotype switching, intercellular communication, single-cell transcriptome

## Abstract

**Background:**

Vascular smooth muscle cell (VSMC) phenotype switching has been preliminarily found in aortic aneurysms. However, two major questions were raised: (1) What factors drive phenotypic switching of VSMCs in aortic aneurysms? (2) What role does VSMC phenotype transformation play in aortic aneurysms? We speculated that the interaction between infiltrated immune cells and VSMCs played a pivotal role in aortic aneurysm expansion.

**Materials and Methods:**

We obtained single-cell transcriptome data GSE155468 that incorporate eight aortic aneurysm samples and three normal aorta samples. A standard single-cell analysis procedure was performed by Seurat (v3.1.2) for identifying the general cell components. Subsequently, VSMCs were extracted separately and re-clustered for identifying switched VSMC phenotypes. VSMC phenotype annotation was relied on the definitions of specific VSMC phenotypes in published articles. Vital VSMC phenotypes were validated by immunofluorescence. Next, identified immune cells and annotated vital VSMC phenotypes were extracted for analyzing the intercellular communication. R package CellChat (v1.1.3) was used for investigating the communication strength, signaling pathways, and communication patterns between various VSMC phenotypes and immune cells.

**Result:**

A total of 42,611 cells were identified as CD4 + T cells, CD8 + T cells, VSMC, monocytes, macrophages, fibroblasts, endothelial cells, and B cells. VSMCs were further classified into contractile VSMCs, secreting VSMCs, macrophage-like VSMCs, mesenchymal-like VSMCs, adipocyte-like VSMCs, and T-cell-like VSMCs. Intercellular communication analysis was performed between immune cells (macrophages, B cells, CD4 + T cells, CD8 + T cells) and immune related VSMCs (macrophage-like VSMCs, mesenchymal-like VSMCs, T-cell-like VSMCs, contractile VSMCs). Among selected cell populations, 27 significant signaling pathways with 61 ligand–receptor pairs were identified. Macrophages and macrophage-like VSMCs both assume the roles of a signaling sender and receiver, showing the highest communication capability. T cells acted more as senders, while B cells acted as receivers in the communication network. T-cell-like VSMCs and contractile VSMCs were used as senders, while mesenchymal-like VSMCs played a poor role in the communication network. Signaling macrophage migration inhibitory factor (MIF), galectin, and C-X-C motif chemokine ligand (CXCL) showed high information flow of intercellular communication, while signaling complement and chemerin were completely turned on in aortic aneurysms. MIF and galectin promoted VSMC switch into macrophage-like phenotypes, CXCL, and galectin promoted VSMCs transform into T-cell-like phenotypes. MIF, galectin, CXCL, complement, and chemerin all mediated the migration and recruitment of immune cells into aortic aneurysms.

**Conclusion:**

The sophisticated intercellular communication network existed between immune cells and immune-related VSMCs and changed as the aortic aneurysm progressed. Signaling MIF, galectin, CXCL, chemerin, and complement made a significant contribution to aortic aneurysm progression through activating immune cells and promoting immune cell migration, which could serve as the potential target for the treatment of aortic aneurysms.

## Highlights

- MIF signaling from T cells promoted the transformation of contractile VSMCs to macrophage-like VSMCs; MIF signaling from VSMCs recruited immune cells (especially B cells) into aortic aneurysms.
- GALECTIN signaling presented the immunomodulatory capacity of macrophage-like VSMCs and macrophages and promoted the formation of macrophage-like VSMCs and T-cell-like VSMCs.
- CXCL signaling promoted VSMC switch into T-cell-like phenotypes and mediated immune cell migration into aortic aneurysms.
- COMPLEMENT and CHEMERIN signaling were turned on in aortic aneurysms, which promoted macrophage migration, thereby aggravating aortic inflammation.

## Introduction

Aortic aneurysm refers to permanent localized dilation of the aorta (expansion ratio > 150% or diameter > 3 cm) (1). Although aortic aneurysms are usually asymptomatic and are diagnosed on physical examination, ruptured aortic aneurysms have a mortality rate of more than 80 percent. Therefore, medical intervention for aortic aneurysm is necessary. Currently, the effective treatments for aortic aneurysm are open surgery repair (OSR) and endovascular aneurysm repair (EVAR), whose indication is an aneurysm diameter over 5.5 cm (2). The application of OSR and EVAR is limited by a large number of complications and a large financial burden. OSR causes significant physical harm to patients, especially for thoracic aortic aneurysm (TAA). However, currently, there is no effective pharmacotherapy for aortic aneurysm, which is due to the misunderstanding of the pathogenesis of aortic aneurysm. Early studies have suggested that the pathological features of aortic aneurysms include loss or apoptosis of VSMCs (3), immune cell infiltration (lymphocytes, neutrophils, dendritic cells, macrophages) (4), extracellular matrix remodeling (5), atherosclerosis (6), and intraluminal thrombus (7). However, none of the clinical drug trials based on these conventional mechanisms has shown the ability to reduce AAA expansion, including MMP inhibitors (doxycycline) (8), antiatherosclerotic drugs (fenofibrate, statins) (9, 10), and antithrombotic drugs (ticagrelor). The failure of these clinical trials suggests that the pathophysiological mechanisms of aortic aneurysm remain poorly studied.

Previous studies indicated that the lack of contractile force caused by VSMC apoptosis or VSMC loss was one of the direct causes of aortic dilatation. Subsequently, emerging studies found that VSMCs in aortic aneurysms underwent phenotypic transformation. Part of VSMCs lost the expression of specific contractile protein and transformed into intermediate state cells which could further transform into macrophage-like VSMCs (expressing macrophage markers MAC2 and CD68) under conditional stimulation (11). Phenotypic transformation of VSMCs is regulated by growth factor (such as connective tissue growth factor, CTGF) (12), non-coding RNA (such as miR-143/145) (13), transcription factor (such as Kruppel-like factor 4, KLF4) (14), and environmental factor (such as lactate) (15).

However, no studies have shown the effect of changes in aortic aneurysm cell composition on phenotypic transformation of VSMCs. We are interested in how VSMCs function and interact with surrounding cells as they undergo phenotypic transformation. The infiltrating immune cells in aortic aneurysms include neutrophils, macrophages, B cells, and T cells, which maintain a chronic inflammatory environment in the aorta. Since the transformed VSMCs also exhibited immune-related phenotypes, we speculated that immune cells interacted with VSMCs during the phenotypic transformation of VSMCs. The interaction of immune cells with target cells depends on a series of receptor–ligand binding, which is a precondition for the activation of immune cells. For example, the interaction between antigen-presenting cells (APC) and T cells depends on the binding of MHC-CD3 and costimulatory receptor CD28-B7. Therefore, we believe that on the one hand, immune cells promote the transformation of VSMCs to immune-related phenotypes; on the other hand, immune-related VSMCs strengthen the function of immune cells and recruit immune cells to infiltrate into the aorta, promoting aortic aneurysm growth. Therefore, the present study investigated the interaction pattern between immune-related VSMC and immune cells in aortic aneurysms, as well as the involved signaling pathway and ligand–receptor pairs, which could be the potential target for the treatment of aortic aneurysms.

## Materials and Methods

### Data Source and Data Pre-processing

Single-cell sequencing data for eight human aortic aneurysm samples (four males, four females) and three human normal aorta samples (one male, two females) were obtained from GSE155468 (16). Patients in dataset GSE155468 aged from 56 to 78 years and the maximum aneurysm diameter ranged from 4.9 cm to 5.8 cm. Of note, 10 of 11 patients were non-Hispanic, nine of 11 patients were white, and 10 of 11 patients had hypertension. Aortic aneurysm samples and normal aorta samples were merged for creating two Seurat objects, respectively, through using Seurat R package (version 3.1.2) (17). Subsequently, data integration was performed between the aortic aneurysm Seurat object and the normal aorta Seurat object through identifying anchors between the two datasets. Cells with less than 200 genes or more than a 10% mitochondria content and genes with less than 10 cells were removed. The count matrix was normalized and scaled by “NormalizeData” function and “ScaleData” function in Seurat, respectively.

### General Cell Type Identification and Vascular Smooth Muscle Cell Phenotype Identification

Top 2000 highly variable genes (HVGs) were calculated through “FindVariableFeatures” function in Seurat, and top 20 principal components were calculated by “RunPCA” function according to the top 2000 HVGs. The t-distributed stochastic neighbor embedding (t-SNE) algorithm with a solution of 0.6 was used for clustering and visualization of all cell clusters. The automatic cell annotation algorithm “SingleR” annotated all cell clusters by using databases “HumanPrimaryCellAtlasData” and “BlueprintEncodeData” as the reference. Background knowledge pertaining to the cellular components of normal aortas and aortic aneurysms was used to assist cell annotation. VSMCs identified by the aforementioned steps were separately extracted, and HVGs and top 20 principal components were recalculated for re-clustering. Because VSMC phenotype transformation is a new concept proposed in recent years and its identification relies on the expression of specific marker genes, SingleR does not work on VSMC phenotypic annotation. In order to recognize marker genes, we calculated differently expressed genes through “FindAllMarkers” function in Seurat. The threshold of log fold-change was set as 0.25, and the Wilcoxon rank sum test was used to test the significance of differences.

### Mice Aortic Aneurysm Model Construction

Male C57/BL6 mice (8–10 weeks old, 25–30 g) were obtained from the Experimental Animal Centre, Shanxi Medical University, China. The mice were anesthetized using ether inhalation. Then, abdominal organs were exposed *via* a median abdominal incision. The abdominal aorta was exposed through reversing colons and intestines. The abdominal aorta was isolated from the inferior vena cava under an optical microscope. Gelatin sponge (1 mm × 1 mm × 5 mm) was soaked in elastase solution (100 mg/ml) and was then covered on the surface of the aorta for 20 min. Subsequently, the abdominal cavity was irrigated with normal saline at 37°C for three times. The muscle layer and skin layer were sutured, respectively. After surgery, the mice recovered on a 37°C warm pad. A feed of 0.2% (v/v) 3-aminopropionitrile fumarate (BAPN) was given to mice to help aortic aneurysm formation. After 21 days, aortic aneurysm was obtained for immunofluorescence staining.

### Immunofluorescence Staining

Briefly, the mice aortic aneurysm was irrigated with normal saline, fixed in 4% paraformaldehyde, and dehydrated in graded ethanol. The sections were immersed in ethylenediaminetetraacetic acid (EDTA) antigen retrieval buffer, and endogenous peroxidase was blocked by 3% H_2_O_2_. After blocking with 3% bovine serum albumin (BSA), the sections were incubated with αSMA antibody (Santa Cruz, sc-32251, 1:200), CD68 antibody (Santa Cruz, sc-20060, 1:200), CD3D antibody (Abcam, ab213362, 1:200), and CD34 antibody (Santa Cruz, sc-7324, 1:200) overnight at 4°C. Secondary antibody was incubated for 50 min at room temperature.

### Analysis of General Intercellular Communication Between Immune Cells and Immune-Related Vascular Smooth Muscle Cells

R package “CellChat” (version 1.1.3) is the latest algorithm that infers intercellular communication from gene expression levels from single-cell transcriptome data (18). Immune cells (incorporating macrophages, CD4 + T cells, CD8 + T cells, and B cells), contractile VSMCs, and immune-related VSMC phenotypes (including mesenchymal-like VSMC, T-cell-like VSMC, and macrophage-like VSMC) were extracted for intercellular communication analysis. The gene expression profile of extracted cells was input for identifying differentially overexpressed ligands and receptors of each cell population. “CellChat” first calculated a probability value of each ligand–receptor interaction, and then the communication probability of each signaling pathway was calculated by summarizing the probability of its subordinate ligand–receptor pairs. The communication probability refers to communication strength. Statistically significant interactions were counted if *p*-value < 0.05. Finally, the number of significant interactions and communication strength were visualized by circ plot.

### Identification of Vital Signaling Pathways and Ligand–Receptor Pairs in the Communication Network

“CellChat” allows researchers to visualize each signaling pathway or ligand–receptor pair between cell groups of interest. Immune cells were considered as source cells and VSMCs as target cells, and then their positions were switched. For vital signaling pathways, the hierarchy plot was first applied for visualizing the network structure among source cells and target cells. Heatmap was plotted for picturing communication probabilities between all cell pairs. Network centrality analysis was performed to investigate the role each cell population played in the signaling pathway. The roles incorporate “Sender,” “Receiver,” “Mediator,” and “Influencer.” The contribution of significant ligand–receptor pairs to the signaling pathway in the communication network was analyzed by calculating the relative ratio of communication strength of single ligand–receptor pair to that of the whole signaling pathway.

### Recognition of Communication Patterns

Except for investigating individual pathways, “CellChat” also allows exploring how multiple cell groups and signaling pathways act in concert to function. To achieve this, “CellChat” outputs the so-called incoming/outgoing communication patterns for uncovering the coordination relationship between cell groups and signaling pathways. In addition, “CellChat” could recognize the similarity between signaling pathways and group similar signaling pathways into groups.

### Exploring Changes of Signaling Pathways and Communication Patterns Between the Aortic Aneurysm and Normal Aorta

Selected cell types from the aortic aneurysm and normal aorta were used for constructing two CellChat objects, respectively. The information flow value of each signaling pathway was first calculated by summarizing all communication probabilities of the signaling pathway. Significant signaling pathways were sorted according to differences in their relative information flow ratio in the inferred networks between the aortic aneurysm and normal aorta. The alterations of incoming/outgoing communication strength were illustrated in two-dimensional diagrams.

### Quantitative Polymerase Chain Reaction

qPCR was used to quantify vital ligands and receptors in signaling MIF, galectin, CXCL, chemerin, and complement. Total RNA was extracted from mice aortic aneurysm tissues using the Tiangen RNA Simple Total RNA Kit (DP419, Tiangen). Subsequently, 1 μg of total RNA was reverse-transcribed using PrimeScript RT Master Mix (RR036A, Takara). Amplification was performed using SYBR Green Premix (RR420A, Takara). NADPH was used as the internal reference for mRNA qPCR. The independent sample *t*-test was used to validate any significant differences of relative expression levels between the aortic aneurysm and normal aorta. *P*-values < 0.05 were considered statistically significant. Primers used in this work are listed in Supplementary Data Sheet 1.

## Results

### Identified Cell Types and Vascular Smooth Muscle Cell Phenotypes in Aortic Aneurysms

**FIGURE 1:**
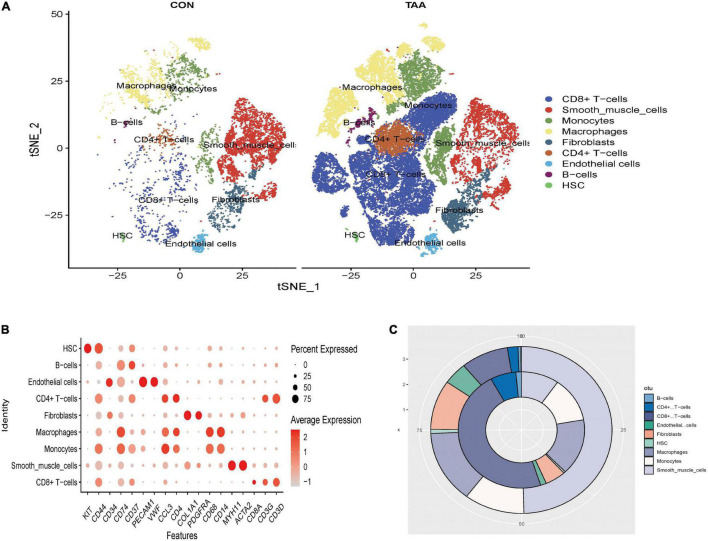
Clustering all cells filtrated from GSE155468. The t-distributed stochastic neighbor embedding (t-SNE) plot of the aligned gene expression of cells from the normal aorta and aortic aneurysms, respectively, showing the distribution of nine cell identities **(A)**. The expression level and expression percentage of marker genes are illustrated on the dot plot **(B)**. The change of proportion of identified cell types was shown in a donut chart **(C)**. The proportion of adipocyte-like VSMCs, mesenchymal-like VSMCs, macrophage-like VSMCs, and T-cell-like VSMCs significantly increased in aortic aneurysms (inner donut), and the proportion of contractile VSMCs dramatically decreased in the normal aorta (outer donut).

A total of 42,611 cells with 20,551 genes remained after unqualified cells and genes were filtrated. Top 2000 HVGs were calculated for clustering (see Supplementary Data Sheet 2). The unsupervised clustering algorithm clustered 42,611 cells into 25 cell populations. Marker genes of each cell cluster could be seen in Supplementary Data Sheet 3. Then the automated reference-based annotation algorithm “SingleR” annotated 25 cell populations as cell types incorporating CD4 + T cells, CD8 + T cells, VSMCs, monocytes, macrophages, fibroblasts, endothelial cells, B cells, and hematopoietic stem cells (HSC) (Figures 1A,B). The percentage of CD4 + T cells, CD8 + T cells, B cells, monocytes, and macrophages increased in aortic aneurysms, while the proportion of VSMCs and fibroblasts significantly decreased (Figure 1C).

**FIGURE 2:**
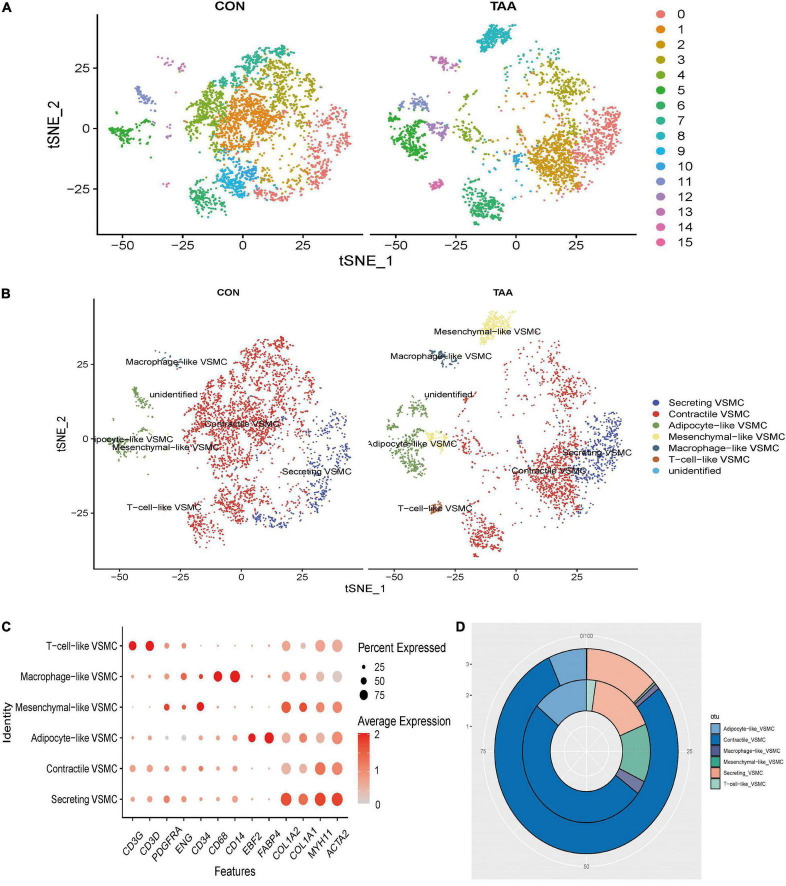
Reanalysis of VSMC subtypes. t-SNE of identified 16 cell clusters **(A)**, which were then annotated as six VSMC phenotypes **(B)**. The expression level and expression percentage of specific marker genes were illustrated on the dot plot **(C)**. The proportion of monocyte, B cells, T cells, and macrophages was increased in aortic aneurysms (inner donut), while the proportion of smooth muscle cells, fibroblasts, and HSC was higher in the normal aorta (outer donut) **(D)**.

Subsequently, all VSMCs were separately extracted and re-clustered into 16 VSMC clusters (Figure 2A). Marker genes of each VSMC cluster could be seen in Supplementary Data Sheet 4. According to the canonical definition of each specific VSMC phenotype (19), we identified six VSMC phenotypes incorporating contractile VSMCs (ACTA2 + MYH11+), secreting VSMCs (ACTA2 + MYH11 + COL1A1 + COL1A2+), mesenchymal-like VSMCs (ACTA2 + MYH11 + CD34 + PDGFRA +), adipocyte-like VSMCs (ACTA2 + MYH11 + FABP4 + EBF2+), macrophage-like VSMCs (ACTA2 + MYH11 + CD14 + CD68+), and T-cell-like VSMCs (ACTA2 + MYH11 + CD3D + CD3G+) (Figures 2B,C). The percentage of secreting VSMCs, mesenchymal-like VSMCs, adipocyte-like VSMCs, macrophage-like VSMCs, and T-cell-like VSMCs significantly increased in aortic aneurysms, whereas contractile VSMCs were reduced (Figure 2D).

### Successful Validation of Immune-Related Vascular Smooth Muscle Cell Phenotypes in the Mice Model of Aortic Aneurysms

**FIGURE 3:**
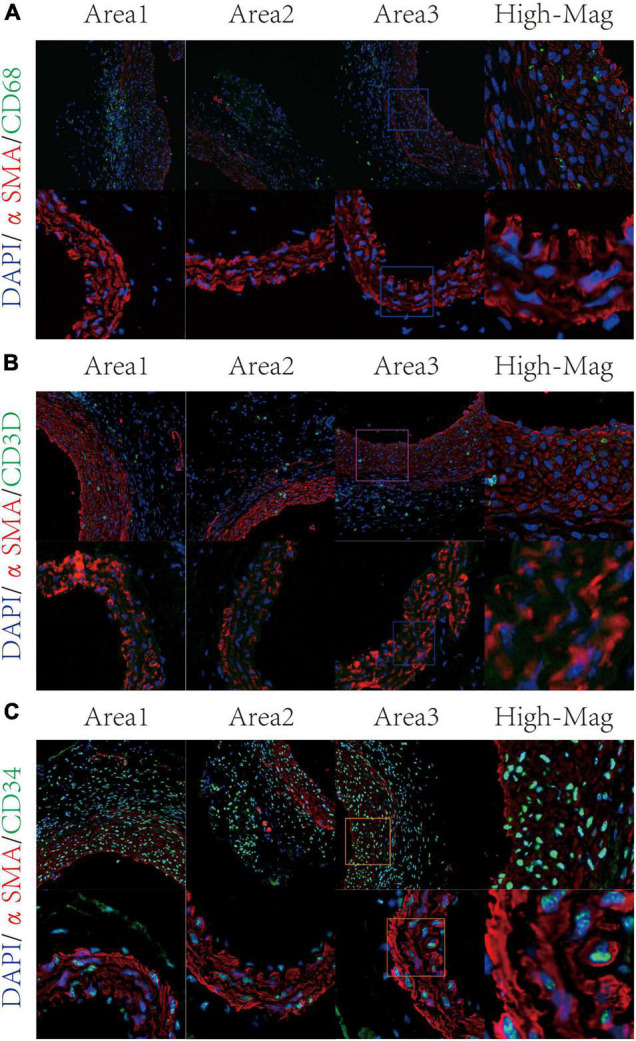
Validation of VSMC subtypes through immunofluorescence staining. Macrophage-like VSMC was labeled by αSMA and CD68 **(A)**. T-cell-like VSMC was labeled by αSMA and CD3D **(B)**; mesenchymal-like VSMC was labeled by αSMA and CD34 **(C)**.

According to published articles, mesenchymal-like VSMCs were prone to switch into several phenotypes such as macrophage-like VSMCs (20). Therefore, we consider mesenchymal-like VSMCs, macrophage-like VSMCs, and T-cell-like VSMCs as immune-related VSMCs. In order to confirm the presence of immune-related VSMC phenotypes in aortic aneurysms, we constructed mice models of aortic aneurysms through periaortic elastase induction. After 21 days, no mice died during the modeling process, and all six mice were successfully modeled (see Supplementary Figure 1). Aortic aneurysm tissues were obtained for double immunofluorescence staining. Macrophage-like VSMCs (αSMA + CD68+) were observed in the tunica media of aortic aneurysms but were not fund in the normal aorta (Figure 3A). T-cell-like VSMCs (αSMA + CD3D+) existed in the tunica media of aortic aneurysm but not in the normal aorta (Figure 3B). Mesenchymal-like VSMCs appeared in both of the aortic aneurysm and normal aorta but demonstrated a much greater proportion in aortic aneurysms (Figure 3C).

### Overview of Intercellular Communication Between Immune Cells and Immune-Related Vascular Smooth Muscle Cells

**FIGURE 4:**
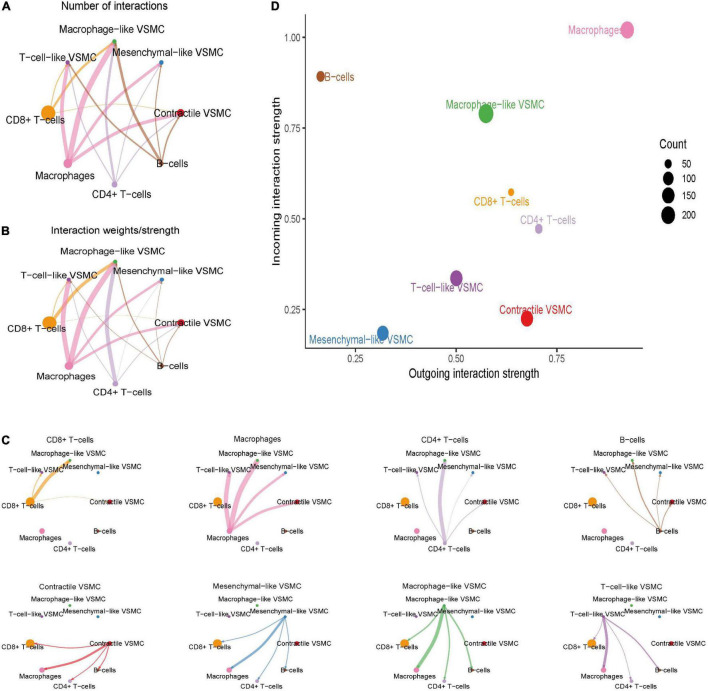
Global cell–cell communication between immune cells and selected VSMC phenotypes. The overall intercellular interaction number **(A)** and weight **(B)** were visualized in the network. The line width represents the intensity. Subsequently, the communication intensity from each immune cell type to all VSMC phenotypes or from each VSMC phenotype to all immune cell types was visualized, respectively **(C)**. The incoming and outgoing communication capacity of each cell type **(D)**.

The overall communication number and weight between immune cells and immune-related VSMCs were quantified and visualized (Figures 4A,B). Among all immune cells, macrophages showed the highest numbers and strengths of interaction with each VSMC phenotypes, especially macrophage-like VSMCs and T-cell-like VSMCs (Figure 4C). CD4 + T cells and CD8 + T cells both demonstrated a strong communication number and strength with macrophage-like VSMCs but weaker communication with other VSMC phenotypes. Nevertheless, B cells showed a weak intercellular communication with all VSMC phenotypes. In brief, macrophages possess the strongest intercellular communication with all VSMC phenotypes.

We subsequently investigated the role of cells in the communication network (Figure 4D). Macrophages and macrophage-like VSMCs both assume the roles of senders and receivers, and they showed the highest communication capability. CD8 + T cells and CD4 + T cells acted more as a sender, rather than a receiver. B cells act primarily as a receiver in the communication network. T-cell-like VSMCs and contractile VSMCs acted as a sender in the communication network, whereas mesenchymal-like VSMCs play a poor role in the communication network.

### Significant Signaling Pathways and Ligand–Receptor Pairs Between Immune Cells and Immune-Related Vascular Smooth Muscle Cells

A total of 61 significant ligand–receptor pairs were identified among immune cells and VSMC phenotypes, which were grouped into 27 signaling pathways incorporating but not limited to MIF, galectin, CXCL, growth arrest-specific gene (GAS), pleiotrophin (PTN), visfatin, CC chemokine ligand (CCL), annexin, and secreted phosphoprotein 1 (SPP1) (see Supplementary Data Sheet 5). We understand this mechanism in terms of the sender/target and signaling pathway, respectively. We first set immune cells as the senders and set VSMCs as the receivers because we speculated that signaling from immune cells to VSMCs promotes phenotype transformation of VSMCs (Supplementary Figure 2A). We found that macrophages sent the most complex signaling pathways, while macrophage-like VSMCs received the most kinds of signaling pathways. Among these significant signaling pathways, MIF–(CD74 + CD44) pairs mediated the strongest communication probability from CD4 + T cells and CD8 + T cells to macrophage-like VSMCs. NAMPT–(ITGA5 + ITGB1) showed the highest communication force from macrophages and B cells to macrophage-like VSMCs. In addition, galectin signaling (LGALS9-CD44/CD45) also played a pivotal role in the communication from macrophages to T-cell-like VSMCs and macrophage-like VSMCs. In conclusion, the formation of macrophage-like VSMCs is associated with MIF, NAMPT, and galectin signaling, and the formation of T-cell-like VSMCs is associated with galectin signaling.

Subsequently, immune cells were set as the receivers and VSMCs were set as the senders because we reckon that signaling from VSMCs to immune cells could explain how immune-related VSMC phenotypes aggravate aortic aneurysm progression (Supplementary Figure 2B). We found that macrophage-like VSMCs were characterized by the maximum number of the signaling pathways sent, and macrophages received the maximum signaling pathways. Through PTN, galectin, and CXCL signaling, macrophage-like VSMCs could communicate with all immune cells, indicating the potential role of modulating immune response. Additionally, CXCL12–CXCR4 pair also connected T-cell-like VSMCs and mesenchymal-like VSMCs with all immune cells. We also noticed the high communication probability of MIF signaling from contractile VSMCs, T-cell-like VSMCs, and macrophage-like VSMCs to B cells, which may be associated with B cell infiltration in aortic aneurysms.

**FIGURE 5:**
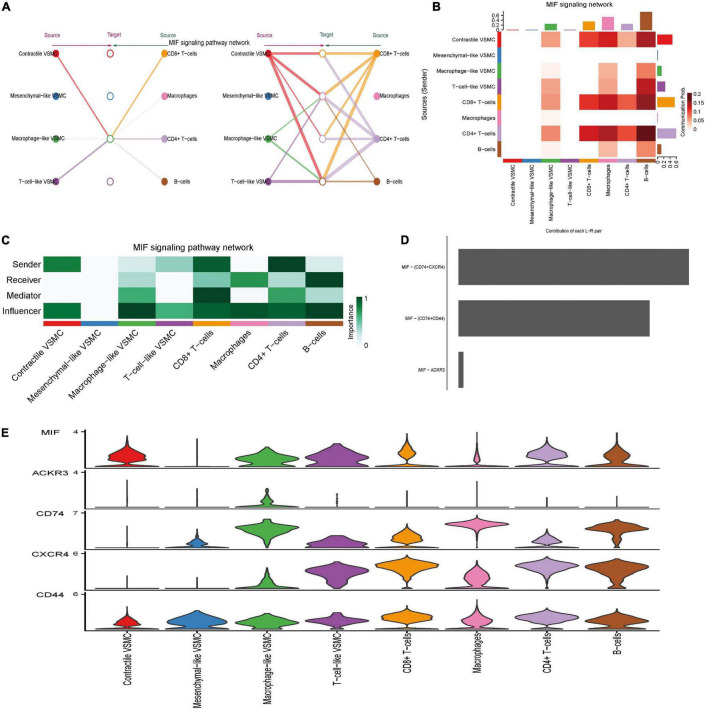
MIF signaling pathway-mediated intercellular communication intensity is shown in the hierarchy plot **(A)** and heatmap **(B)**. Network center score showed the role each cell type played, including sender, receiver, mediator, and influencer **(C)**. Contribution of each ligand–receptor pair to MIF signaling **(D)**. Expression level of every ligand and receptor gene in each cell type **(E)**.

Among 27 significant signaling pathways, MIF, galectin, and CXCL were the signaling pathways with the highest communication strength (see Supplementary Data Sheet 6) and seem to play a significant role in immune regulation. Thus, we showed all detailed intercellular communication mediated by them and the role each cell played in these pathways. The MIF pathway mediated intercellular communication among multiple distinct cells and formed a sophisticated communication network (Figures 5A,B). The hierarchy plot indicated that macrophage-like VSMCs and immune cells were the primary target of the MIF pathway. CD8 + T cells and CD4 + T cells were the principal secreting cells, and importantly, macrophages merely secreted MIF. CD8 + T cells obtained the highest mediator score, which indicated that CD8 + T cells act as a gatekeeper to control the communication flow in the inferred MIF signaling network (Figure 5C). Of note, macrophage-like VSMCs acquired the highest influencer score, indicating that macrophage-like VSMCs possess a high capacity of the influencing information flow. Ligand MIF primarily combined with receptors CD74, CD44, and CXCR4 (Figures 5D,E).

Distinct from the redundant and complex signaling structures of MIF, galectin signaling demonstrated its simple inferred network structure. Only two cell groups, macrophages and macrophage-like VSMCs, acted as the sender cells, while all cells were the receivers (especially CD4 + T cells and CD8 + T cells) (Supplementary Figures 3A,B). Macrophages also took on the role of a mediator and influencer (Supplementary Figure 3C). Of note, only one ligand (LGALS9) was paired with three receptors (CD44, CD45, and HAVCR2), and LGALS9–CD44 pairs seem to make the greatest contribution (Supplementary Figures 3D,E).

The senders of CXCL signaling were macrophages, mesenchymal-like VSMCs, T-cell-like VSMCs, and macrophage-like VSMCs, while the receivers were mainly T-cell-like VSMCs, CD4 + T cells, CD8 + T cells, and B cells (Supplementary Figures 4A–C). CXCL singling was mainly mediated by CXCL12–CXCR4 and CXCL16–CXCR6 pairs (Supplementary Figure 4D). Macrophages and immune-related VSMCs (mesenchymal-like, T-cell-like, and macrophage-like VSMCs) secreted CXCL12/16 that were then received by T cells, B cells, and T-cell-like VSMCs. T-cell-like VSMCs were also characterized by playing a mediator role. The level of CXCL12/16 in macrophage-like VSMCs is as high as that in macrophages, while the level of CXCR4/6 is similar between T cells and T-cell-like VSMCs, which indicated the functional similarity between macrophages and macrophage-like VSMCs and similarity between T cells and T-cell-like VSMCs (Supplementary Figure 4E).

### Coordinated Communication Patterns

In addition to investigating the role of individual signaling pathways in intercellular pathways, it is of great significance to explore how cells and significant pathways work in concert. The outgoing communication patterns were first studied, in which all cells were considered as secreting cells (Supplementary Figure 5A). The analysis revealed that contractile VSMCs, mesenchymal-like VSMCs, and macrophage-like VSMCs shared the pattern #1 that incorporated signaling pathways such as GAS, PTN, tumor necrosis factor-like weak inducer of apoptosis (TWEAK), platelet-derived growth factors (PDGF), and fibroblast growth factor (FGF). Outgoing signaling of macrophages and B cells were characterized by pattern #2 including but not limited to galectin. CXCL, visfatin, SPP1, progranulin (GRN), epidermal growth factor (EGF), and tumor necrosis factor (TNF). Meanwhile, CD4 + T cells and CD8 + T cells both concentrated on outgoing communication pattern #3, driven by pathways including MIF, transforming growth factor beta (TGFb), interferon-II (IFN-II), protease-activated receptors (PARs), and CD40. On the other hand, when considered as target cells/receivers, contractile VSMCs, mesenchymal-like VSMCs, and T-cell-like VSMCs showed the same pattern #1 that included PTN, visfatin, SPP1, TWEAK, GRN, and PDGF (Supplementary Figure 5B). Macrophage-like VSMCs and macrophages demonstrated the immune-related incoming pattern #2 such as CCL, annexin, IFN-II, CD40, and complement, indicating their functional similarity in the immune signaling pathway. In brief, through analyzing communication patterns, we learned that distinct cell groups could share mostly overlapping signaling pathways, such as the similarity between macrophage-like VSMCs and macrophages.

### Alterations of Intercellular Communication Patterns Between the Normal Aorta and Aortic Aneurysm

**FIGURE 6:**
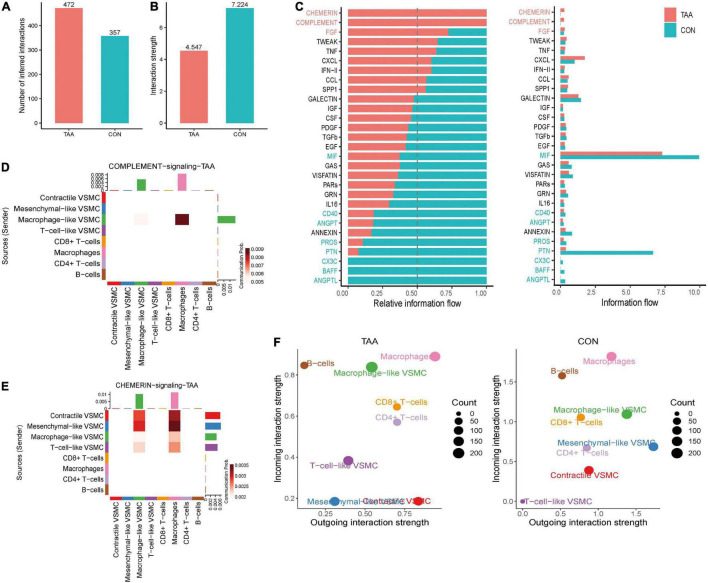
Alterations of intercellular communication patterns between the aortic aneurysm and normal aorta. The number of interactions increased **(A)**, but the mean interaction strength decreased in aortic aneurysms **(B)**. Relative information flow of the significant signaling pathway in the normal aorta and aortic aneurysm **(C)**. Sources and targets of signaling complement **(D)** and chemerin **(E)**. Alteration of incoming/outgoing communication ability of each cell type between the aortic aneurysm and normal aorta **(F)**.

The aforementioned analysis investigated the intercellular communication from a global perspective. Furthermore, the changes in communication patterns between the normal aorta and aortic aneurysm samples could interpret the relationship between intercellular communication and aortic aneurysm progression. Altogether, the number of significant interactions between selected VSMC phenotypes and immune cells was more numerous in aortic aneurysms than in the normal aorta (472 vs. 357) (Figure 6A). However, the average interaction strength was reduced in aortic aneurysms compared with the normal aorta (Figure 6B).

Second, we compared the information flow between the aortic aneurysm and normal aorta (Figure 6C). In aortic aneurysms, relative information flow of signaling chemerin, FGF, complement, TWEAK, hepatocyte growth factor (HGF), TNF, CXCL, IFN-II, CCL, and SPP1 were elevated and signaling chemerin and complement were completely turned on, whereas the relative information flow of signaling galectin, insulin-like growth factor (IGF), colony-stimulating factor (CSF), PDGF, TGFb, EGF, MIF, and GAS were decreased and signaling (C-X3-C motif chemokine) CX3C, B-cell activating factor (BAFF), and angiopoietin-like protein (ANGPTL) were completely turned off in aortic aneurysms. Therefore, we speculated that signaling chemerin and complement play a pivotal role in promoting VSMC phenotype switching in aortic aneurysms. We visualized signaling chemerin and complement by heatmap (Figures 6D,E). In complement signaling, macrophage-like VSMCs acted as a sender, and the message was received by macrophages. For macrophage-like VSMCs, complement signaling can be regarded as the autocrine signaling pathway to a certain extent. Regarding to chemerin signaling (RARRES2-CMKLR1), contractile VSMCs and mesenchymal-like VSMCs seem to have the highest communication strength as a signaling source, and macrophage-like VSMCs and VSMCs received signaling. As CMKLR1 mediates the migration of macrophages and dendritic and NK cells (21), we speculated that chemerin signaling from VSMCs could active immune cell migration and aggravating aortic aneurysm progression.

We also compared the intensity of incoming and outgoing communication of each cell population between the aortic aneurysm and normal aorta, which was projected in a two-dimensional plot (Figure 6F). In brief, the incoming communication strength of macrophage-like VSMCs and T-cell-like VSMCs significantly increased, whereas that of mesenchymal-like VSMCs and contractile VSMCs significantly decreased. On the other hand, the outgoing communication strength of T-cell-like VSMCs and contractile VSMCs was dramatically enhanced, while that of macrophage-like VSMCs, mesenchymal-like VSMCs, and B cells was reduced.

Finally, we compared the difference in incoming signaling patterns and outgoing signaling patterns between the aortic aneurysm and normal aorta. Although the overall outgoing signaling was significantly decreased in macrophage-like VSMCs, the intensity of CXCL, FGF, chemerin, complement, and PROS increased (Supplementary Figure 6A). According to incoming signaling, GAS, annexin, chemerin, CD40, PROS, and ANGPT were significantly elevated (Supplementary Figure 6B). Of note, the overall outgoing signaling and incoming signaling of T-cell-like VSMCs both dramatically increased, including a variety of pathways.

### Validation of Expression Levels of Vital Signaling Pathways in Aortic Aneurysm

**FIGURE 7:**
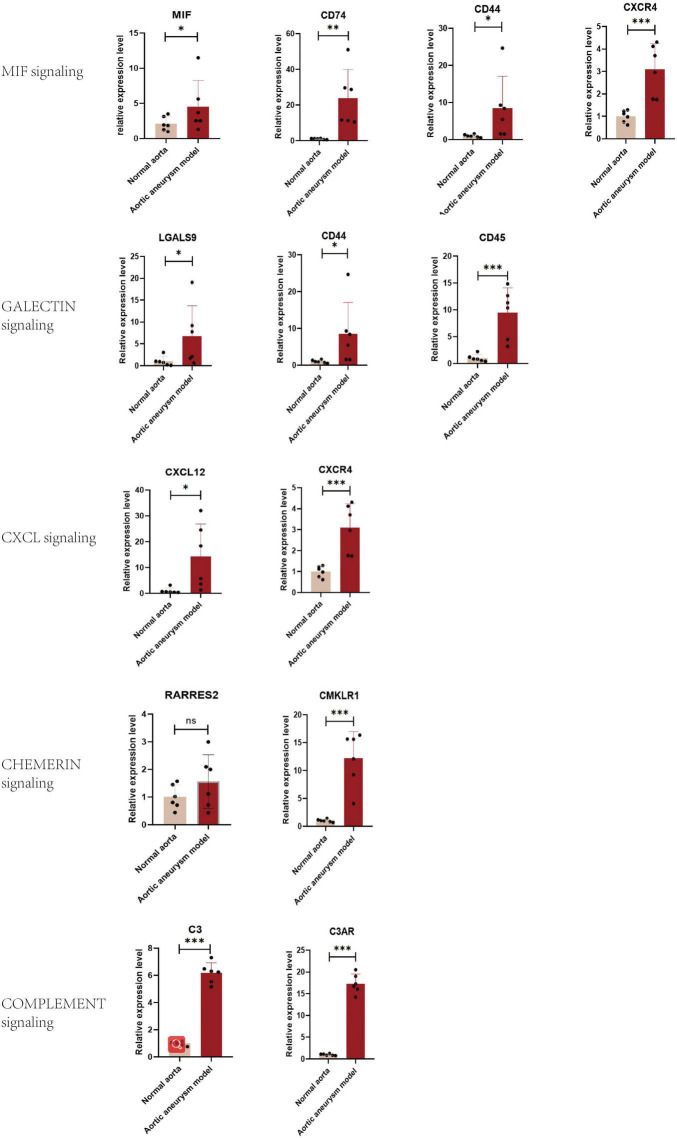
Validation of the expression level of significant ligand–receptor pairs of signaling MIF, galectin, CXCL, chemerin, and complement by qPCR. *P < 0.05, **P < 0.001, ***P < 0.0001.

Quantitative polymerase chain reaction was performed for quantifying relative expression of significant ligand–receptor pairs of signaling MIF, galectin, CXCL, chemerin, and complement (Figure 7). For MIF signaling, the expression level of all ligand and receptors were upregulated: MIF = 2.142 (*p* < 0.05), CD74 = 23.82 (*p* < 0.05), CD44 = 8.476 (*p* < 0.05), and CXCR4 = 4.398 (*P* < 0.001). For galectin signaling, all ligands and receptors were overexpressed LGALS9 = 6.739 (*P* < 0.05), CD44 = 8.476 (*p* < 0.05), CD45 = 9.463 (*p* < 0.001). For CXCL signaling, the ligand–receptor pairs were overexpressed: CXCL12 = 14.28 (*p* < 0.05) and CXCR4 = 3.101 (*p* < 0.05). However, for chemerin signaling, the ligand RARRES2 showed a non-significant upregulation: RARRES2 = 1.560 (*p* = 0.113) and CMKLR1 = 12.17 (*p* < 0.001). Finally, the C3-C3AR pair in complement signaling was also overexpressed, C3 = 6.174 (*p* < 0.0001) and C3AR = 17.28 (*p* < 0.0001).

## Discussion

In the healthy mature artery, VSMCs present a terminally differentiated and quiescent state termed as contractile VSMCs, which are characterized by little capacity of proliferation and migration but high capacity of contraction (22). Contractile VSMCs exist in the tunica media and contractile to confront the expansion force when large amounts of blood are pumped from the heart into the aorta, whereas once as injury occurred or the external environment changed, contractile VSMC loss its contractile markers such as ACTA2 and MYH11 and switched into an intermediate state. VSMCs in the intermediate state could transform into distinct phenotypes by receiving distinct stimulation (20). Some VSMC phenotypes, such as macrophage-like VSMCs, showed the inherent characteristics of immune cells such as phagocytosis and secreting chemokines (23). Therefore, it is natural to doubt whether an intimate communication exists between immune cells and VSMCs. The present study aimed to address the concern.

We found that the communication intensity between macrophages and VSMCs was the highest among all immune cells, while macrophage-like VSMCs showed the highest intercellular communication with immune cells. Similarly, the communication intensity between macrophages and macrophage-like VSMCs is also noticeable. It suggests that macrophages and macrophage-like VSMCs and their interaction may play a pivotal role in the progression of aortic aneurysms.

When analyzing communication between immune cells and VSMCs, we set two directions: from immune cells to VSMCs and from VSMCs to immune cells. We raised a hypothesis that signaling from immune cells to VSMCs acts as a trigger activating VSMC switching into immune-related phenotypes. On the other hand, signaling from immune-related VSMCs to immune cells exerts a chemotaxis function that attracts more immune cells to migrate to aorta lesions. In the whole analysis results, many signaling pathways showed special importance, but we considered MIF, galectin, CXCL, chemerin, and complement to be the most important.

Among all the significant signaling pathways identified from immune cells to VSMCs, MIF signaling was the one that shines the brightest. MIF was characterized by the highest information flow. We found that MIF–(CD44-CD74) pairs mediated signaling from CD4 + T cells and CD8 + T cells to macrophage-like VSMCs. On the other hand, MIF–(CD74 + CXCR4) mediated signal transmission from contractile VSMCs, T-cell-like VSMCs, and macrophage-like VSMCs to B cells Numerous studies have shown that MIF possesses the function of recruiting and activating macrophages through combining with CD74 and CXCR2 (24–26) and promote normal cells to acquire an inflammatory phenotype by interacting with the receptor CD74 (27). MIF binds to CD74 + CXCR4, which promotes B-cell migration (28). Therefore, in aortic aneurysms, MIF from T cells plays a role in promoting the transformation of VSMCs to macrophage-like VSMCs, while MIF from VSMCs recruits immune cells (especially B cells) into aortic aneurysms. As previous studies showed that galectin 9–CD44 interaction is in favor of stability and function of adaptive regulatory T cells (29), galectin 9–CD45 inhibits naive B-cell activation (30), and can inhibit CD4 + T-cell expansion and suppress Th1 effector function (31). Hence, we speculate that macrophage-like VSMCs and macrophages both exert an immunomodulatory capacity through the galectin signaling pathway. The recipients of CXCL signals are mainly T cells, B cells, and T-cell-like VSMCs. Previous studies have shown that T and B cells infiltrated in abdominal aortic aneurysms are CXCR4-positive and are recruited by CXCL12-positive stromal cells (32). CXCL16-CXCR6 also mediates the recruitment of lymphocytes and peripheral blood mononuclear cells (33, 34). Therefore, in aortic aneurysms, mesenchymal-like, macrophage-like, and T-cell-like VSMC can recruit circulating lymphocytes through the CXCL signaling pathway, exacerbating the aortic immune response. Furthermore, CXCL may promote VSMC switch into T-cell-like phenotypes since T-cell-like VSMC is the main receiver of CXCL signaling.

Signaling complement and chemerin did not exist in the normal aorta but were turned on in aortic aneurysm. In complement signaling, macrophage-like VSMCs secreted C3 and then C3 combined with receptors in macrophages and macrophage-like VSMCs including C3AR1, ITGAM, ITGAX, and ITGB2. ITGAM (integrin αM), a surface marker in mononuclear macrophages, is also termed as CD11b. ITGAM acts as an adhesion molecule and mediated the migration of circulating monocytes/macrophages; thus, ITGAM deficiency could ameliorate aortic aneurysm expansion (35). Similarly, C2AR1, ITGAX (CD11c), and ITGB2 (CD18) also promote the infiltration of macrophages in immune-mediated disease (36–38). Hence, complement signaling exerts a harmful effect that promotes aortic aneurysm expansion through mediating macrophage infiltration and migration. Regarding chemerin signaling, all VSMC phenotypes secreted chemerin (encoded by RARRES2), and then chemerin was received by chemerin chemokine-like receptor 1 (CMKLR1) in macrophages. Chemerin–CMKLR1 pair stimulated macrophage transformation to the M1 (proinflammatory) subtype *via* the p-Akt/CEBPα axis (39) and mediated the migration of macrophages and dendritic cells (40). Collectively, signaling complement and chemerin mainly promoted macrophage migration, thereby aggravating aortic inflammation.

## Conclusion

Extensive intercellular communications exist between multiple VSMC phenotypes and immune cells, as macrophages and macrophage-like VSMCs mediated the highest communication intensity.

Signaling MIF, galectin, and CXCL showed high information flow of intercellular communication, while signaling complement and chemerin were completely turned on in aortic aneurysms. MIF and galectin promoted VSMC switch into macrophage-like phenotypes, and CXCL and galectin promoted VSMC transform into T-cell-like phenotypes. MIF, galectin, CXCL, complement, and chemerin all mediated the migration and recruitment of immune cells into aortic aneurysms.

## Data Availability Statement

The original contributions presented in the study are included in the article/Supplementary Material, further inquiries can be directed to the corresponding author.

## Ethics Statement

The animal study was reviewed and approved by Ethics Committee of the Second Hospital of Shanxi Medical University.

## Author Contributions

GC and HD designed the study and wrote the manuscript. GC and ZL conducted the data analysis. GC, RG, and XX completed the animal experiment. RZ and HD revised the manuscript and figures. HD supervised the whole study. All authors contributed to the article and approved the submitted version.

## Conflict of Interest

The authors declare that the research was conducted in the absence of any commercial or financial relationships that could be construed as a potential conflict of interest.

## Publisher’s Note

All claims expressed in this article are solely those of the authors and do not necessarily represent those of their affiliated organizations, or those of the publisher, the editors and the reviewers. Any product that may be evaluated in this article, or claim that may be made by its manufacturer, is not guaranteed or endorsed by the publisher.

## Supplementary Material

Supplementary Figure 1 Successful construction of mice model of aortic aneurysm.

Supplementary Figure 2 The significant signaling (ligand-receptor pairs) from immune cells to VSMC subtypes **(A)** and from VSMC phenotypes to immune cells **(B)**.

Supplementary Figure 3 GALECTIN signaling pathway mediated intercellular communication intensity was showed in hierarchy plot **(A)** and heatmap **(B)**. Network center score showed the role each cell type played, including sender, receiver, mediator, and influencer **(C)**. Contribution of each ligand-receptor pair to GALECTIN signaling **(D)**. Expression level of every ligand and receptor gene of GALECTIN signaling in each cell type **(E)**.

Supplementary Figure 4 CXCL signaling pathway mediated intercellular communication intensity was showed in hierarchy plot **(A)** and heatmap **(B)**. Network center score showed the role each cell type played, including sender, receiver, mediator, and influencer **(C)**. Contribution of each ligand-receptor pair to CXCL signaling **(D)**. Expression level of every ligand and receptor gene of CXCL signaling in each cell type **(E)**.

Supplementary Figure 5 The outgoing communication patterns **(A)** and incoming communication patterns **(B)** of each cell type was visualized by river plot.

Supplementary Figure 6 Alteration of outgoing communication ability **(A)** and incoming communication ability **(B)** of every significant signaling pathway in each cell type between normal aorta and aortic aneurysm.
