# Supplementary figures and images for: Deciphering the Intercellular Communication Between Immune Cells and Altered Vascular Smooth Muscle Cell Phenotypes in Aortic Aneurysm From Single-Cell Transcriptome Data
Source: Front Cardiovasc Med. 2022 Jun 28;9:936287. doi: 10.3389/fcvm.2022.936287 (PMC9273830; doi:10.3389/fcvm.2022.936287)

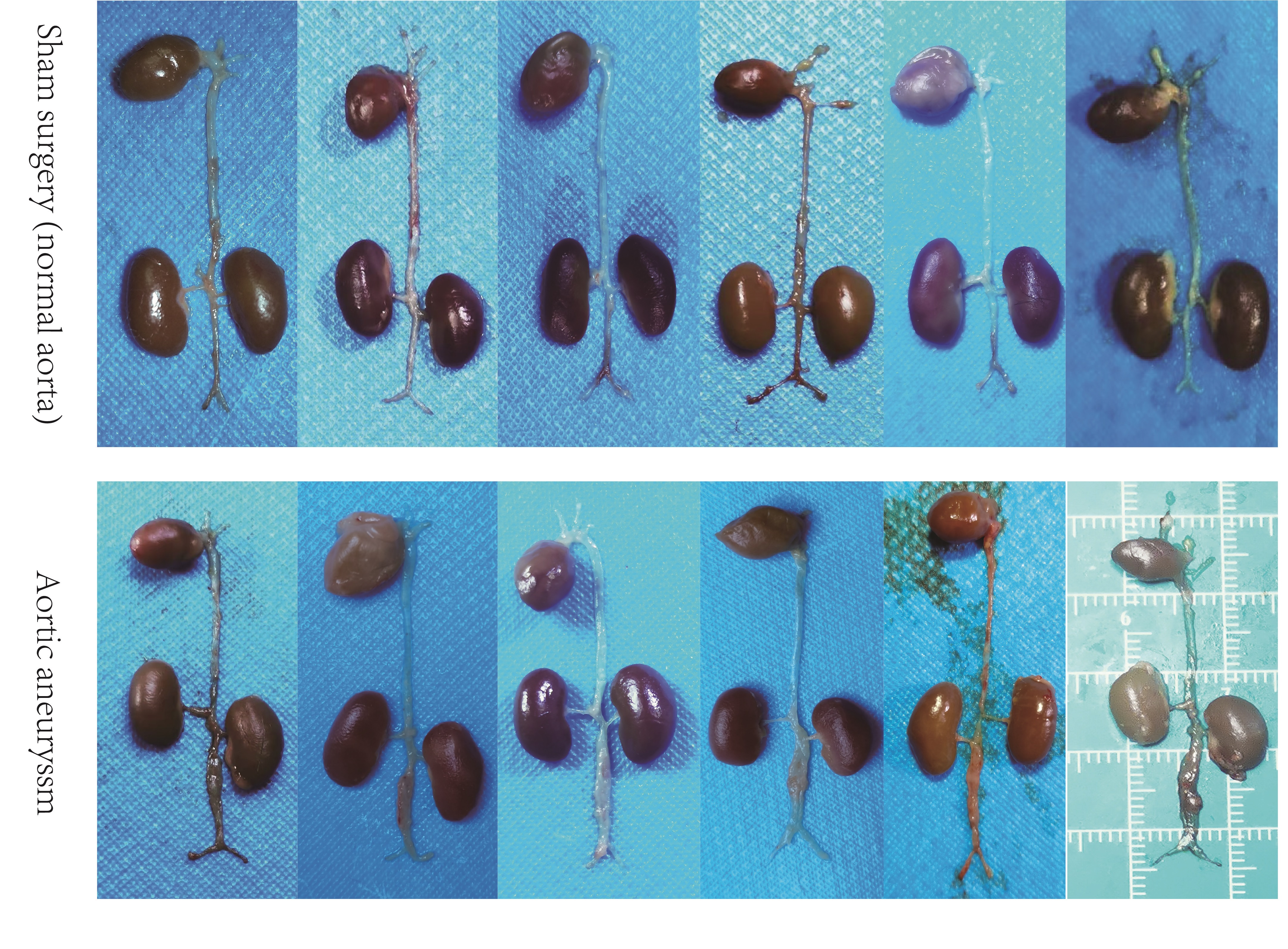

Supplement: Supplementary Figure 1 — Successful construction of mice model of aortic aneurysm. [file Image_1.JPEG]

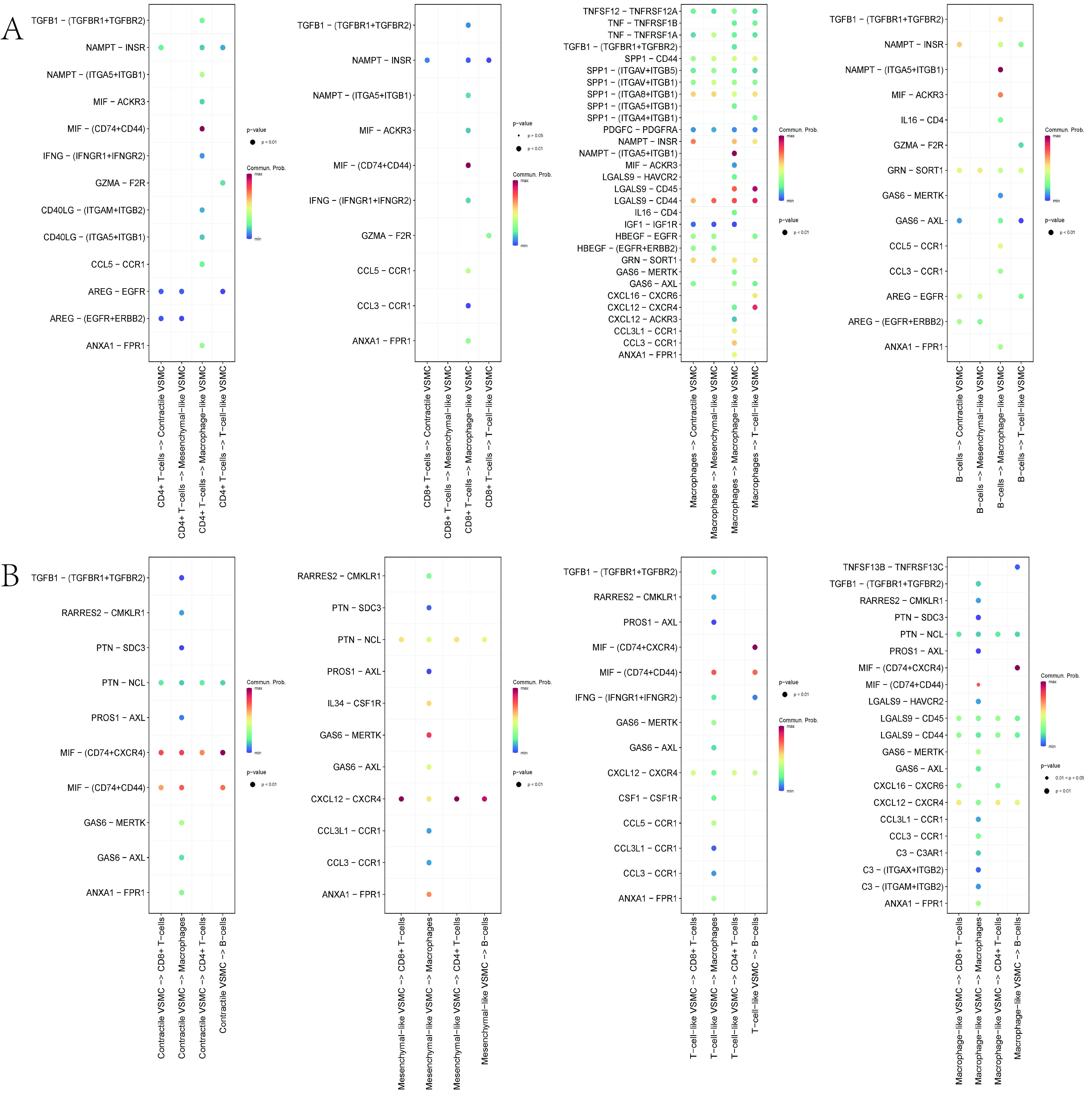

Supplement: Supplementary Figure 2 — The significant signaling (ligand-receptor pairs) from immune cells to VSMC subtypes (A) and from VSMC phenotypes to immune cells (B). [file Image_2.JPEG]

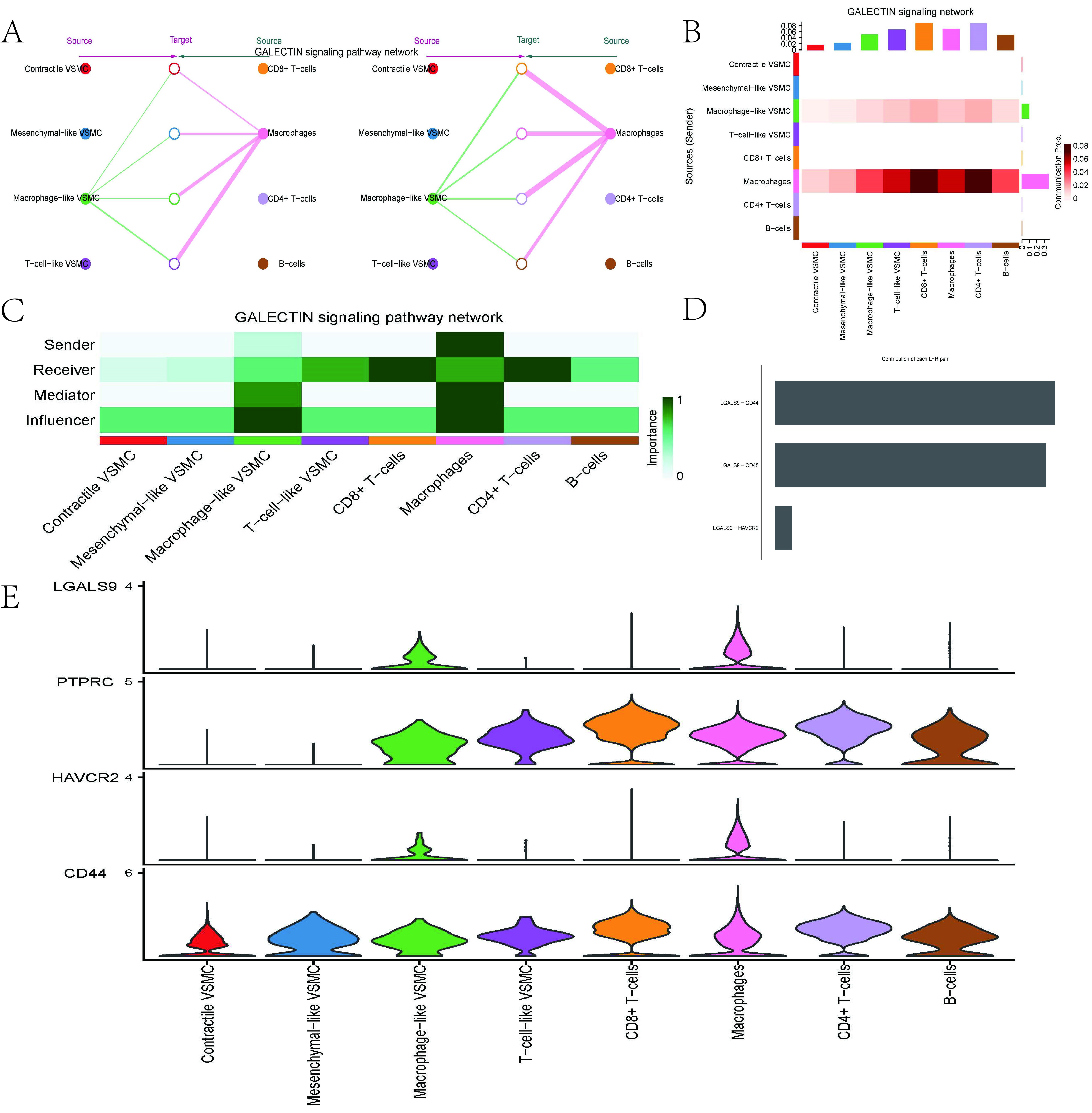

Supplement: Supplementary Figure 3 — GALECTIN signaling pathway mediated intercellular communication intensity was showed in hierarchy plot (A) and heatmap (B). Network center score showed the role each cell type played, including sender, receiver, mediator, and influencer (C). Contribution of each ligand-receptor pair to GALECTIN signaling (D). Expression level of every ligand and receptor gene of GALECTIN signaling in each cell type (E). [file Image_3.JPEG]

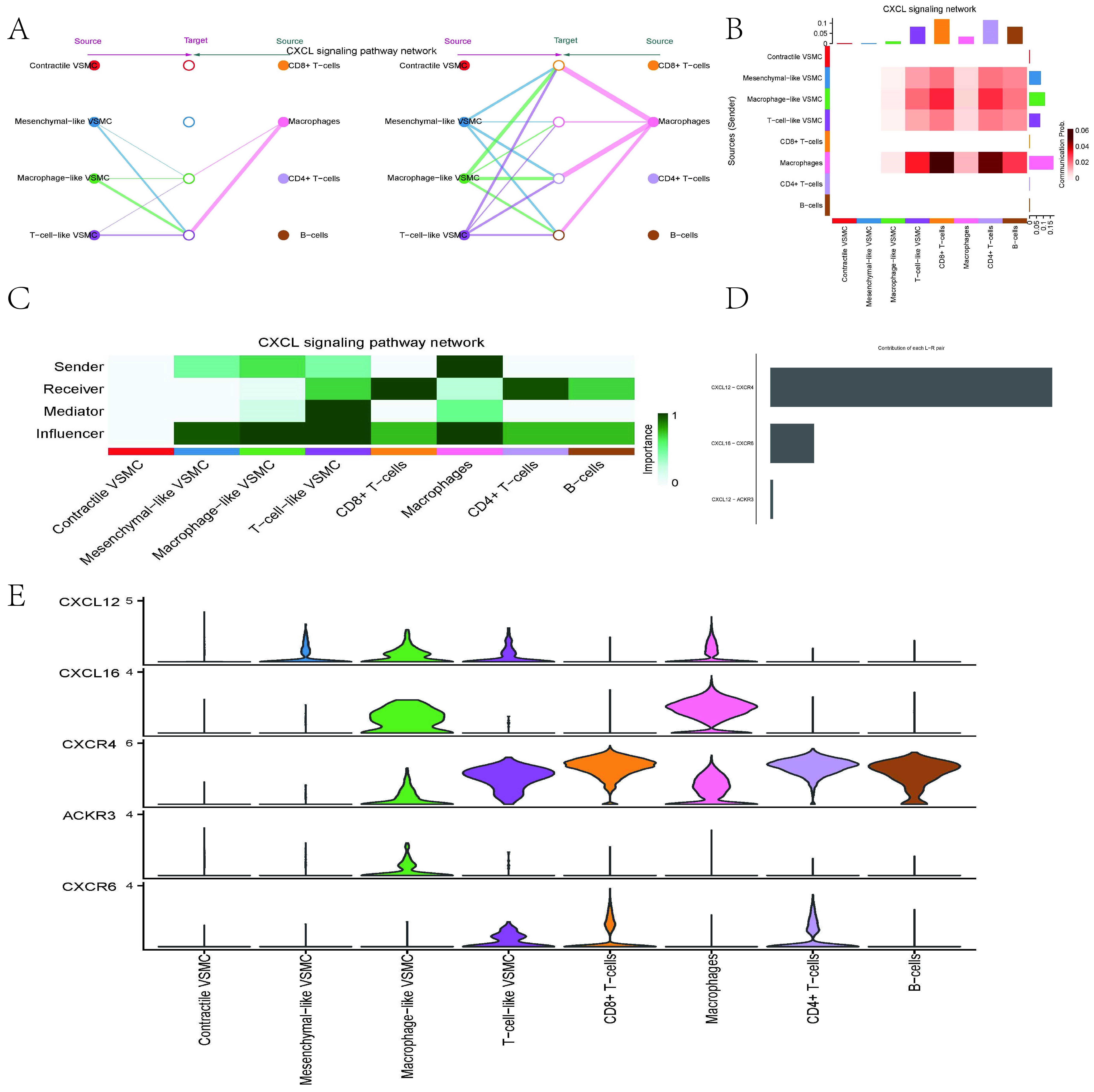

Supplement: Supplementary Figure 4 — CXCL signaling pathway mediated intercellular communication intensity was showed in hierarchy plot (A) and heatmap (B). Network center score showed the role each cell type played, including sender, receiver, mediator, and influencer (C). Contribution of each ligand-receptor pair to CXCL signaling (D). Expression level of every ligand and receptor gene of CXCL signaling in each cell type (E). [file Image_4.JPEG]

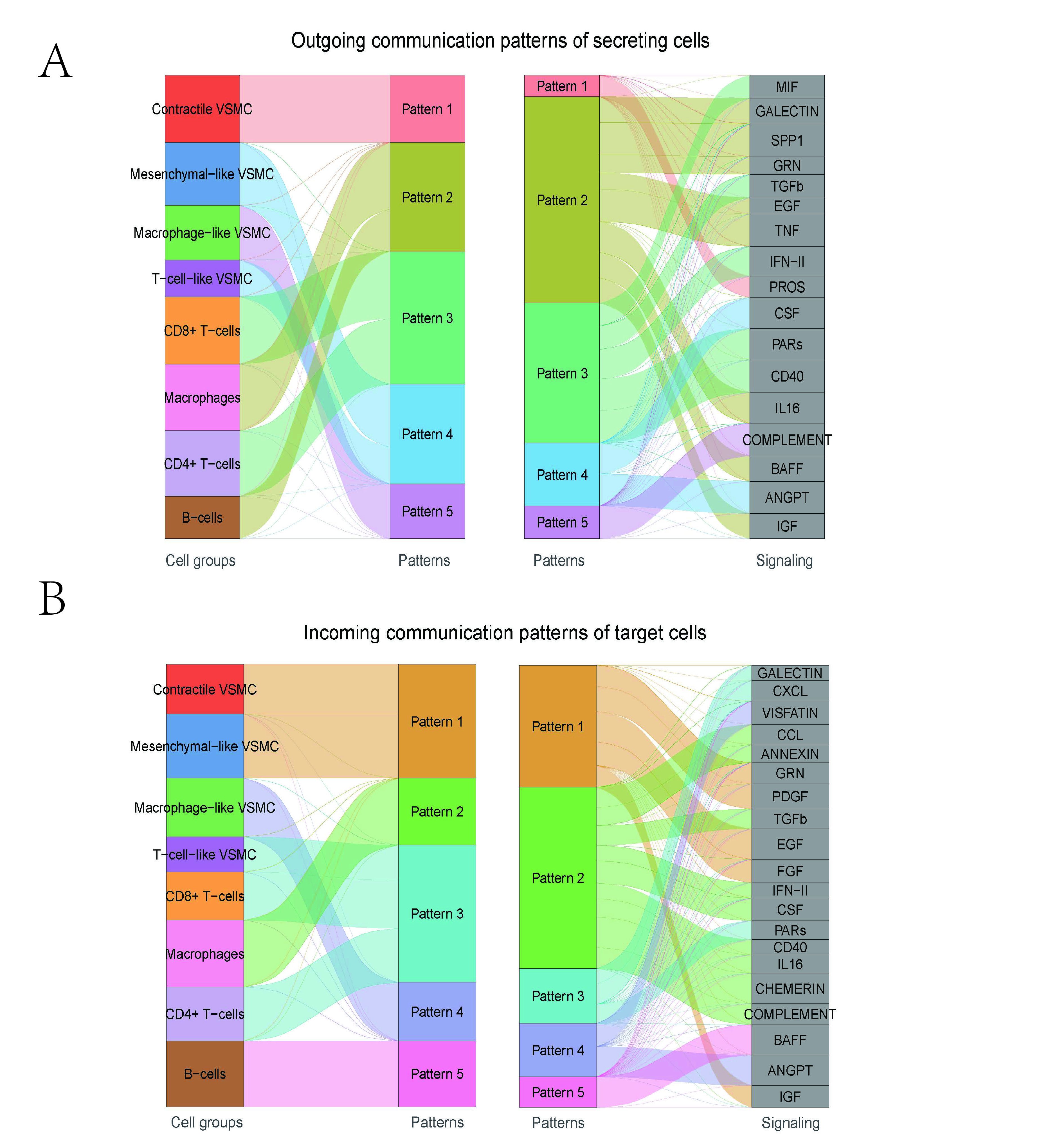

Supplement: Supplementary Figure 5 — The outgoing communication patterns (A) and incoming communication patterns (B) of each cell type was visualized by river plot. [file Image_5.JPEG]

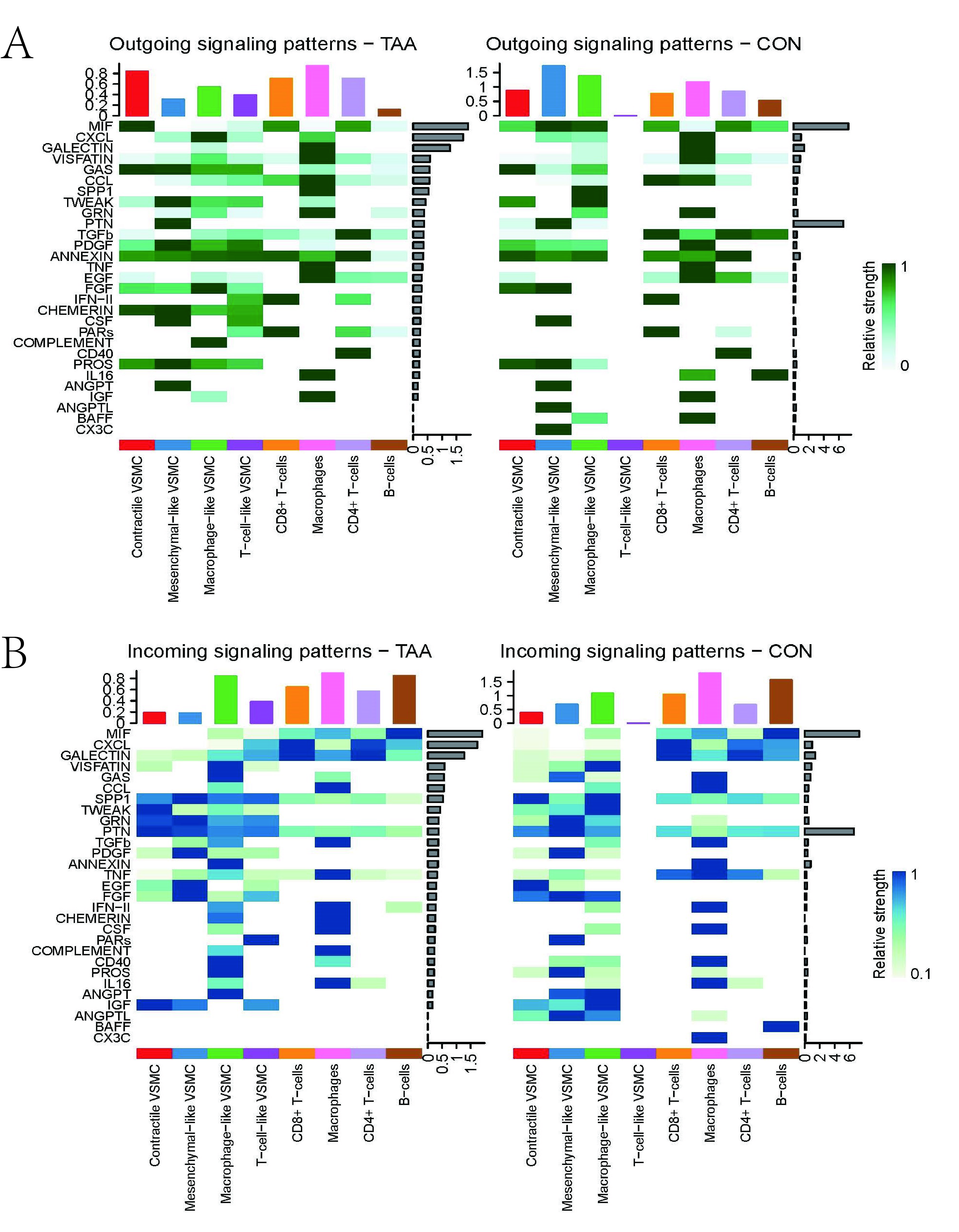

Supplement: Supplementary Figure 6 — Alteration of outgoing communication ability (A) and incoming communication ability (B) of every significant signaling pathway in each cell type between normal aorta and aortic aneurysm. [file Image_6.JPEG]
